# Survival analysis of patients with invasive extramammary Paget disease: implications of anatomic sites

**DOI:** 10.1186/s12885-018-4257-1

**Published:** 2018-04-10

**Authors:** Haijun Yao, Minkai Xie, Shibo Fu, Jianhua Guo, Yubing Peng, Zhikang Cai, Yueqing Jiang, Dachao Zheng, Zhong Wang

**Affiliations:** Department of Urology, Shanghai 9th People’s Hospital, Shanghai Jiao Tong University, School of Medicine, 639 Zhizaoju Rd, Shanghai, 200011 People’s Republic of China

**Keywords:** Extramammary Paget disease (EMPD), Anatomic sites, Survival analysis, Surveillance, epidemiology, and end results (SEER)

## Abstract

**Background:**

Extramammary Paget disease (EMPD) is a rare malignant dermatosis with poorly defined outcomes. We investigated clinical characteristics of invasive EMPD at different anatomic sites and by subject demographics to determine prognostic factors for overall survival (OS).

**Methods:**

All patient data were collected from the Surveillance, Epidemiology, and End Results (SEER) program, 1973–2013, of the U.S. National Cancer Institute. Patients with invasive EMPD of skin, vulva/labia, vagina, scrotum/penis, or other sites were included. After excluding patients with unknown radiation status, data of 2001 patients were analyzed. Primary endpoint was EMPD mortality by anatomic sites. Independent variables included patients’ demographic data, concurrent malignancy (ie, non-EMPD related cancers), tumor size, distant metastasis, and surgery and/or radiation or not.

**Results:**

Multivariate regression analysis showed that mortality was significantly higher in patients with vaginal EMPD than in patients with vulvar/labial EMPD (adjusted hazard ratio [aHR] = 3.26, *p* < 0.001). Patients with distant metastasis had higher mortality than those without (aHR = 3.36, *p* < 0.001). Patients who received surgery had significantly lower mortality than those who did not receive surgery (aHR = 0.77, *p* = 0.030), and those treated with radiation had significantly higher mortality than those who did not receive radiation (aHR = 1.60, *p* = 0.002). Older age was associated with significantly increased mortality (aHR = 1.09, *p* < 0.001), and mortality was significantly higher in males than in females (aHR = 1.42, *p* = 0.008).

**Conclusions:**

In conclusion, among EMPD patients, mortality is higher in patients with vaginal EMPD than in those with vulvar/labial EMPD and higher in those who are older, those with concurrent malignancy or distant metastasis. Mortality is also higher in males than in females. Surgery is a protective factor and radiation is a risk factor for OS. Greater understanding of EMPD clinical characteristics, and considering EMPD in differential diagnosis of chronic genital and perianal dermatoses may provide support for early EMPD diagnosis and definitive surgical treatment.

## Background

Extramammary Paget disease (EMPD) is a rare cutaneous adenocarcinoma that targets mainly genital and perianal skin, as well as other cutaneous sites that have abundant apocrine glands [1]. The cell morphology and histology of EMPD is the same as that of mammary Paget disease (PD) of the nipple [2]. These two related skin diseases were first identified in the late nineteenth century and differ mainly by anatomic site [3, 4]. While PD is found almost exclusively in women (male cases are less than 1%, sometimes associated with prostate cancer), invasive EMPD is found in men and women but is more common in women [5]. The most common anatomic sites at which EMPD may arise are the vulva, including labia as part of the vulva, the vagina, or the penis or scrotum and perianal region. Historically, the incidence rates for EMPD have generally been highest for the vulva anatomic site except for a higher incidence of primary skin EMPD in 1978, 1979, and 1994 [6]. Primary skin includes skin of the face, scalp and neck, trunk, and limbs. Although it is a rare disease, EMPD represents about 21% of primary scrotal cancers, which is increasing by 3.2% per year [7]. However, it is unknown why EMPD cases of the scrotum and other anatomic sites are increasing [8].

The histogenesis and pathogenesis are also reported to be different between the two types of Paget disease, but this has been debated considerably between authors. Nevertheless, both diseases present as chronic eczematous cutaneous disease with relatively slow-growing lesions, and both are associated with underlying malignancies [1]. Skin biopsy is typically able to differentiate EMPD from other chronic dermatoses; and primary EMPD and EMPD secondary to an underlying malignancy are differentiated using immunohistochemistry techniques [9]. Almost all cases of mammary PD is associated with underlying breast cancer, and about 1% to 4% of female breast carcinoma will have PD of the nipple, areola, and surrounding skin [10]. EMPD is associated with various other adenocarcinomas such as adenocarcinoma of the digestive system, genitourinary adenocarcinoma or other internal malignancy; some authors have suggested that the location of the underlying malignancy is associated with the anatomic site of EMPD [1].

The prognosis for mammary PD depends on disease stage at diagnosis, lymph node metastasis or not, and the presence or absence of underlying breast carcinoma, which is predictive of higher mortality risk [10]. The five-year survival rates for PD range from 93% to 94% and drop to 82%to 91% at 10 years. The prognosis for EMPD is also worsened by the presence of a concurrent malignancy (ie, not EMPD-related), with mortality rates as high as 46% when underlying malignancy is present compared with an 18% mortality rate without underlying malignancy [1]. In the study by Herrel et al. [8], survival was lower in men with EMPD with distant metastases and primary tumors in the perianal anatomic region. Primary EMPD generally has a better prognosis than secondary cancer, resulting from metastasis of the primary EMPD, and it is clinically critical to differentiate between the two using immunohistochemistry and a panel of appropriate antibodies, although not all underlying malignancies may be identified [9]. In general, however, outcomes are not well defined for this rare disease and EMPD remains an elusive entity lacking both widespread clinician awareness and understanding.

Given the incomplete understanding of EMPD, especially the lack of well-defined outcomes, discrepancies in reporting mortality rates, lack of information on gender and racial incidence, and reasons for increasing incidence, we aimed to investigate the clinical characteristics of invasive EMPD at different anatomic sites and subjects’ demographic profiles to determine prognostic factors for overall survival (OS).

## Methods

### Data source

All data for the present study were from the Surveillance, Epidemiology, and End Results (SEER) Program, 1973–2013, of the National Cancer Institute, DCCPS, Surveillance Research Program, Surveillance Systems Branch [6]. SEER is a nationally representative, longitudinal survey conducted in the United States and statistical data were made available to other researchers in April 2016, based on the November 2015 submission. We obtained permission from the National Cancer Institute, USA, to access the research data file in the SEER program for research purposes only (reference number:12041-Nov2016). All SEER data are de-identified and data analysis for research purposes does not require approval of the Internal Review Board or informed consent by participating subjects.

### Study population

The data of patients diagnosed with invasive EMPD were extracted from the SEER registry for inclusion in analysis. Patients with missing data or unknown radiation status were excluded. A total of 2001 patients diagnosed with invasive EMPD (HISTO3V = 8542 & HST_STGA = 1, 2, 4, 8) were extracted from the SEER database according to codes of the International Classification of Diseases for Oncology (ICD-O) for anatomic sites, as follows: vulva (PRIMSITE = C519); skin (PRIMSITE = C440-C449); penis or scrotum (PRIMSITE = C600-C602, C608-C609, C632); labia (PRIMSITE = C510-C512, C518); vagina (PRIMSITE = C529); other sites (PRIMSITE = any other coding). The “skin” categories identified through the ICD-O codes included eyelid, lip, external auricular canal, other parts of the face, scalp and neck, trunk, upper limb and shoulder, lower limb and hip, overlapping lesion of skin, and skin not otherwise specified.

### Study variables

The main endpoints of the present study were incidence of EMPD, mortality, and OS evaluated by anatomic site. OS was calculated from the day of diagnosis to the date of death from any cause. Independent variables evaluated for each case included patient demographics (age at diagnosis, sex, marital status, race/ethnicity, living in a high latitude state or not), concurrent malignancy, tumor size, distant metastasis, treatment performed (surgery and/or radiotherapy or not), and survival status. All coding and rules in the present study followed guidelines established by the SEER program [6].

### Statistical analysis

Continuous variables such as age are presented as means and standard deviations (mean ± SD) and categorical variables are presented as counts and percentages. Mortality rates were calculated per 10,000,000 population, and direct age adjustment was made according to the 2000 U.S. standard population, as described in the SEER study [6]. The Kaplan-Meier curve with log-rank tests was performed to compare OS between patients with EMPD at different anatomic sites. Univariate and multivariate Cox proportional hazards regression models were constructed to analyze factors associated with survival in patients with EMPD. Two-sided *P* values of < 0.05 were considered statistically significant. Statistical analyses were performed using the statistical software package SAS software version 9.4 (SAS Institute Inc., Cary, NC, USA).

## Results

### Subjects’ baseline demographic and clinical characteristics

Table 1 shows baseline demographic and clinical characteristics of the included patients. A total of 2301 patients with EMPD of any anatomic sites were identified in the SEER database 1973–2013. Among all included patients, 2019 had invasive tumors. After excluding patients whose radiation status was not known, the final study population was 2001 patients. Among these included patients, the mean age was 71.7 years, with 680 (34.0%) males and 1321 females (66%), and a majority of white race (*n* = 1603; 80.1%). Anatomic sites included 54.2% with vulva or labial EMPD, 23.2% with skin EMPD, 16.4% with penis or scrotum EMPD, 0.2% with vagina EMPD, and 5.9% with other sites. Patients with EMPD of any anatomic sites, but not vulva, labial, skin, penis, scrotum, vagina, were all grouped as “Other.”

**Table 1:** Subjects’ baseline demographic and clinical characteristics

Variables	All patients (*n* = 2001)
Anatomic sites (%)	
Vulva or labial	1085 (54.2)
Skin	465 (23.2)
Penis or scrotum	329 (16.4)
Vagina	4 (0.2)
Other sites	118 (5.9)
Concurrent malignancy = Yes (%)	620 (31.0)
Tumor size (%)	
Less than 2 cm	316 (15.8)
More than 2 cm	620 (31.0)
Unknown	1065 (53.2)
Distant metastasis = Yes (%)	46 (2.3)
Surgery = Yes (%)	1771 (88.5)
Radiation = Yes (%)	121 (6.0)
Age (mean ± SD)	71.7 (11.3)
Gender = Male (%)	680 (34.0)
Race (%)	
White	1603 (80.1)
Black	16 (0.8)
Others	356 (17.8)
Unknown	26 (1.3)
High latitude state = Yes (%)	662 (33.1)

Most patients (88.5%) had received surgery. (Table 1).

### Incidence rates among EMPD anatomic sites

**Fig. 1:**
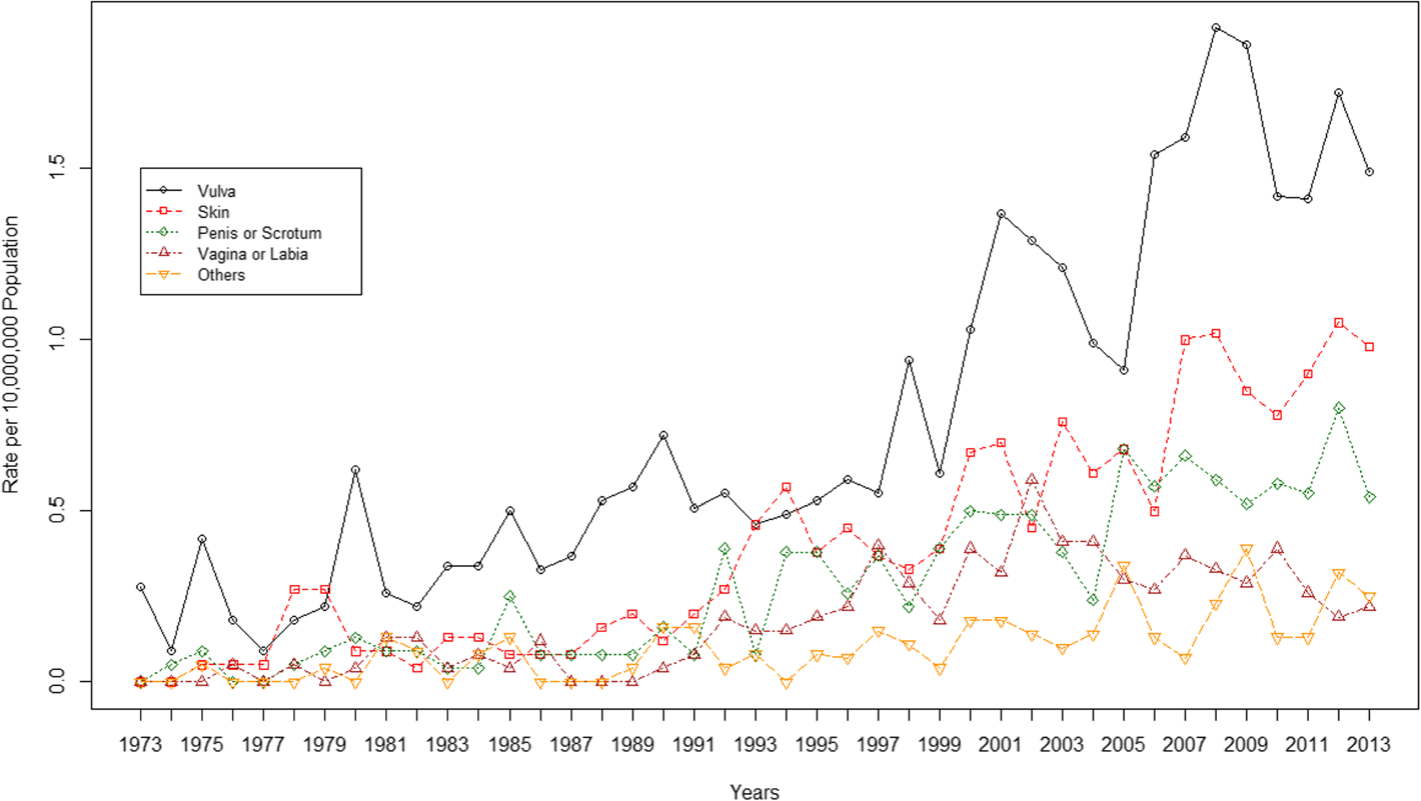
Age-standardized incidence rates of EMPD at different anatomical sites during the period 1973–2003, using 2000 U.S. standard population as reference

The age-standardized incidence rates of different EMPD anatomical sites during the study period 1973–2003 (with 2000 U.S. standard population as reference) are shown in Fig. 1. The vulva or labial anatomic site had the highest incidence rates in general, but during 1978–1979, the highest incidence rate was shown for skin EMPD. Before 1990, incidence rates were similar for EMPD anatomic sites of skin, penis or scrotum, vagina, and other sites (Fig. 1).

### Kaplan-Meier analyses for overall survival according to EMPD anatomic sites

**Fig. 2:**
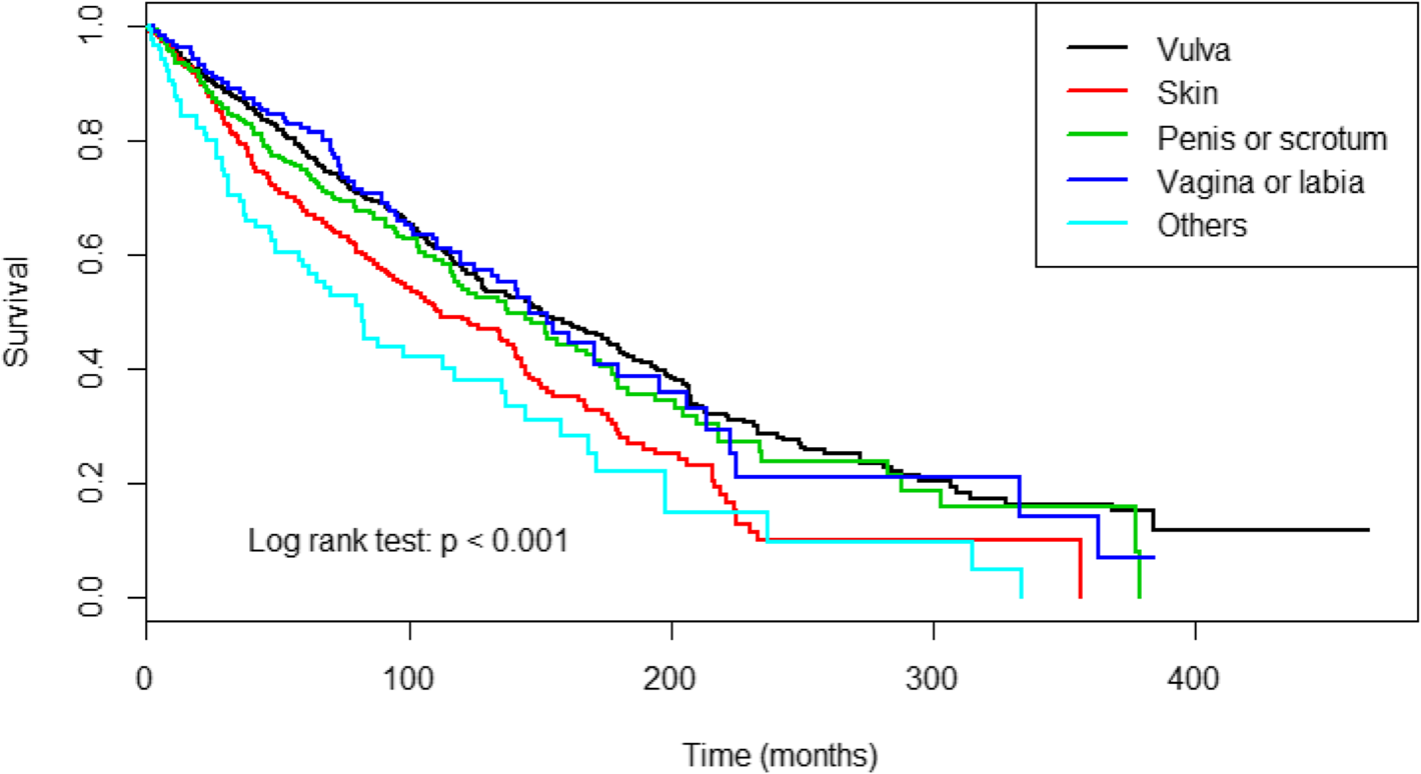
Kaplan-Meier curve depicts survival rates between different EMPD anatomical sites

Figure 2 presents results of Kaplan-Meier curves comparing survival times between different anatomic sites. A total of 892 (44.6%) patients died during the study period. The median survival time was 65 months (IQR: 27–121 months). Overall survival was significantly higher in patients with EMPD of vulva or labial than in those with skin, penis or scrotum, vagina, and other sites (all *p* < 0.05). (Fig. 2).

### Cox proportional hazards models for mortality

Table 2 shows the results of univariate and multivariate Cox proportional hazards regression analysis for factors associated with mortality. Univariate analysis found that mortality was significantly higher among patients with EMPD of the skin and other sites than in those with EMPD of the vulva/labial reference site (skin: HR = 1.48, *p* < 0.001; other sites: HR = 1.99, *p* < 0.001). Mortality was also significantly higher among patients with concurrent malignancy compared with those without other malignancy (HR = 1.59, p < 0.001), and significantly higher in patients with distant metastasis than in those without (HR = 3.49, *p* < 0.001). Patients who received surgery had significantly lower mortality than those without surgery (HR = 0.49, *p* < 0.001); and patients receiving radiation had significantly higher mortality compared with those without radiation (HR = 2.37, *p* < 0.001). Older age was also associated with significantly increased mortality (HR = 1.09, *p* < 0.001). Mortality was also significantly higher in males than in females (HR = 1.35, *p* < 0.001), and significantly lower in patients of other races than in those of white race (HR = 0.79, *p* = 0.010) (Table 2).

**Table 2:** Univariate and multivariate regression analysis of factors associated with mortality in EMPD patients

Variables	HR (95% CI)	*p* value	aHR (95% CI)	*p* value
Anatomic sites				
Vulva or labial	reference		reference	
Skin	***1.48 (1.27, 1.73)***	***<0.001***	0.95 (0.75, 1.20)	0.677
Penis or scrotum	1.14 (0.95, 1.38)	0.171	0.83 (0.60, 1.15)	0.260
Vagina	2.64 (0.73, 9.60)	0.969	***3.26 (2.03, 5.23)***	***<0.001***
Others	***1.99 (1.53, 2.62)***	***<0.001***	1.36 (0.97, 1.90)	0.071
Concurrent malignancy				
No	reference		reference	
Yes	***1.59 (1.37, 1.83)***	***<0.001***	1.15 (0.99, 1.34)	0.058
Tumor size				
Less than 2 cm	reference		reference	
More than 2 cm	1.01 (0.83, 1.24)	0.887	0.92 (0.76, 1.11)	0.377
Distant metastasis				
No	reference		reference	
Yes	***3.49 (2.27, 5.35)***	***<0.001***	***3.36 (2.27, 4.98)***	***<0.001***
Surgery				
No	reference		reference	
Yes	***0.49 (0.39, 0.61)***	***<0.001***	***0.77 (0.61, 0.97)***	***0.030***
Radiation				
No	reference		reference	
Yes	***2.37 (1.83, 3.06)***	***<0.001***	***1.60 (1.18, 2.15)***	***0.002***
Age	***1.09 (1.08, 1.10)***	***<0.001***	***1.09 (1.08, 1.10)***	***<0.001***
Gender				
Female	reference		reference	
Male	***1.35 (1.17, 1.55)***	***<0.001***	***1.42 (1.10, 1.85)***	***0.008***
Race				
White	reference		reference	
Black	0.72 (0.38, 1.34)	0.295	0.65 (0.28, 1.51)	0.313
Others	***0.79 (0.66, 0.94)***	***0.010***	***0.81 (0.67, 0.97)***	***0.026***
High latitude state				
No	reference		reference	
Yes	1.06 (0.92, 1.21)	0.427	0.98 (0.85, 1.13)	0.803

Unknown data for tumor size and race are not shown on the table.

Boldface with italics indicates significant effect. *p*-value < 0.05

After adjusting for associated factors, multivariate regression analysis showed that mortality was significantly higher in patients with EMPD of the vagina than in patients with vulva or labial EMPD (aHR = 3.26, *p* < 0.001). Mortality was also significantly higher in patients with distant metastasis than in those without distant metastasis (aHR = 3.36, p < 0.001). Patients who received surgery had a significantly lower mortality risk than those who did not receive surgery (aHR = 0.77, *p* = 0.030), and those treated with radiation had significantly higher mortality risk compared with those who did not receive radiation (aHR = 1.60, *p* = 0.002). Older age was associated with significantly increased mortality (aHR = 1.09, *p* < 0.001), and mortality was significantly higher in males than in females (aHR = 1.42, *p* = 0.008), and significantly lower in patients of other races than in those of white race (aHR = 0.81, *p* = 0.026) (Table 2).

Table 3 shows the results of univariate and multivariate regression analysis of factors associated with mortality in EMPD patients by radiation treatment. For patients without radiation treatment, multivariate regression analysis showed that the mortality was significantly higher in patients with EMPD of the vagina and others anatomic sites than in patients with vulva or labial EMPD (aHR = 3.20, *p* < 0.001; aHR = 1.61, *p* = 0.005, respectively). Mortality was also significantly higher in patients with distant metastasis than in those without distant metastasis (aHR = 3.43, *p* < 0.001). Patients who received surgery had a significantly lower mortality risk than those who did not receive surgery (aHR = 0.70, *p* = 0.004). Older age was associated with significantly increased mortality (aHR = 1.09, *p* < 0.001), and mortality was significantly higher in males than in females (aHR = 1.50, *p* = 0.003) (Table 3)).

**Table 3:** Univariate and multivariate regression analysis of factors associated with mortality in EMPD patients by radiation treatment

	Without radiation treatment				With radiation treatment			
Variables	HR (95% CI)	*p* value	aHR (95% CI)	*p* value	HR (95% CI)	*p* value	aHR (95% CI)	*p* value
Anatomic sites								
Vulva or labial	reference		reference		reference		reference	
Skin	***1.48 (1.26, 1.75)***	***<0.001***	0.97 (0.77, 1.24)	0.833	0.80 (0.47, 1.34)	0.391	0.62 (0.24, 1.65)	0.342
Penis or scrotum	1.10 (0.90, 1.34)	0.337	0.78 (0.56, 1.08)	0.134	0.95 (0.49, 1.84)	0.888	0.81 (0.26, 2.47)	0.709
Vagina	1.90 (0.47, 7.71)	0.369	***3.20 (1.71, 5.97)***	***<0.001***	***7.26 (3.89, 13.53)***	***<0.001***	***5.76 (2.34, 14.19)***	***<0.001***
Others	***1.96 (1.46, 2.64)***	***<0.001***	***1.61 (1.15, 2.25)***	***0.005***	1.03 (0.52, 2.04)	0.925	0.68 (0.27, 1.73)	0.421
Concurrent malignancy								
No	reference		reference		reference		reference	
Yes	***1.56 (1.34, 1.82)***	***<0.001***	1.14 (0.97, 1.33)	0.107	***1.57 (1.01, 2.44)***	***0.043***	1.21 (0.70, 2.07)	0.498
Tumor size								
Less than 2 cm	reference		reference		reference		reference	
More than 2 cm	1.01 (0.82, 1.24)	0.916	0.91 (0.75, 1.11)	0.337	1.61 (0.86, 3.01)	0.133	1.17 (0.54, 2.53)	0.691
Distant metastasis								
No	reference		reference		reference		reference	
Yes	***3.11 (1.88, 5.14)***	***<0.001***	***3.43 (2.13, 5.52)***	***<0.001***	***2.80 (1.59, 4.92)***	***<0.001***	***2.71 (1.21, 6.10)***	***0.016***
Surgery								
No	reference		reference		reference		reference	
Yes	***0.50 (0.39, 0.65)***	***<0.001***	***0.70 (0.55, 0.89)***	***0.004***	0.83 (0.51, 1.35)	0.455	0.88 (0.48, 1.63)	0.688
Age	***1.09 (1.08, 1.10)***	***<0.001***	***1.09 (1.08, 1.10)***	***<0.001***	***1.06 (1.03, 1.09)***	***<0.001***	***1.06 (1.03, 1.09)***	***<0.001***
Gender								
Female	reference		reference		reference		reference	
Male	***1.35 (1.17, 1.56)***	***<0.001***	***1.50 (1.15,1.95)***	***0.003***	0.81 (0.53, 1.24)	0.328	1.14 (0.47, 2.76)	0.771
Race								
White	reference		reference		reference		reference	
Black	0.69 (0.32, 1.48)	0.338	1.15 (0.45, 2.91)	0.770	0.49 (0.20, 1.22)	0.125	0.43 (0.15, 1.20)	0.108
Others	***0.82 (0.68, 0.98)***	***0.032***	0.86 (0.71, 1.05)	0.132	0.54 (0.26, 1.14)	0.107	***0.44 (0.22, 0.91)***	***0.026***
High latitude state								
No	reference		reference		reference		reference	
Yes	1.09 (0.94, 1.26)	0.251	1.06 (0.91, 1.22)	0.456	0.70 (0.44, 1.12)	0.140	***0.52 (0.30, 0.90)***	***0.019***

Unknown data for tumor size and race are not shown on the table.

Boldface with italics indicates significant effect. *p*-value < 0.05

For patients with radiation treatment, the results showed that the mortality was significantly higher in patients with EMPD of the vagina than in patients with vulva or labial EMPD (aHR = 5.76, *p* < 0.001). Mortality was also significantly higher in patients with distant metastasis than in those without distant metastasis (aHR = 2.71, *p* = 0.016). Older age was associated with significantly increased mortality (aHR = 1.06, *p* < 0.001), and mortality was significantly lower in patients of other races than in those in white races (aHR = 0.40, *p* = 0.026), and significantly lower in patients who live in high latitude state than those who did not (aHR = 0.52, *p* = 0.019) (Table 3)).

## Discussion

In the present study, we compared incidence rates, overall survival and mortality between different anatomic sites of EMPD. The EMPD incidence rates were highest for vulvar or labial EMPD except during 1978–1979 and 1994, when skin EMPD had the highest incidence. Mortality was significantly higher in patients with vaginal EMPD than in patients with vulvar or labial EMPD and higher in patients with distant metastasis. Patients who received radiotherapy had higher mortality than those who did not receive radiation, while patients receiving surgery had significantly lower mortality, making surgery a protective factor while radiation was a risk factor. However, the benefit of protective surgery was only observed in the group of patients who did not receive radiation. Older age was associated with significantly increased mortality, and mortality was significantly higher in males than in females and significantly lower in patients of races other than white or black.

### Overall survival and mortality between anatomic sites

Results of other studies of EMPD agree in general with our findings relative to survival. Herrel et al. [8], who also used the SEER database, showed that primary perianal location, presence of distant metastases, and advanced age were predictors of poor survival in male patients. Perianal EMPD was included in “other” anatomic sites in the SEER study, which showed poorer survival for patients with EMPD of “other” anatomic sites, and those with distant metastases and advanced age. However, results for “other” anatomic sites were non-significant in the present study.

Although in our study and others, patients with vulvar/labial EMPD was the most common among all anatomic sites, the OS in these patients was not higher than that associated with all other anatomic sites. It showed significantly increased mortality for vaginal EMPD compared to vulvar/labial EMPD here. The difference in mortality between patients with vulvar/labial and vaginal EMPD cannot be explained using the SEER data, but other studies offer possible explanations. Some authors suggest that high grade lesions of vulvar EMPD may extend into the vagina and concurrent cervical carcinoma is highly possible [11, 12], which suggests a possible reason for vaginal EMPD having a higher mortality than vulvar EMPD in the present study.

### Concurrent malignancy

In the present study, we can only present associations between concurrent malignancy (non-EMPD-related cancers), distant metastases, and mortality. However, it is of interest to investigate links between EMPD and underlying malignancy. Previous study results suggest that EMPD is associated with concurrent malignancies that may relate directly to the anatomic site of EMPD [1, 13]. For example, EMPD is associated with underlying cutaneous adnexal adenocarcinoma in up to 50% of patients, which is consistent with a higher mortality rate than that found in patients without adnexal adenocarcinoma [8]. Another study of patients with invasive EMPD, using SEER data from 1973 to 2008, identified a long-term increased risk of secondary EMPD malignancy and, in most cases, the malignancy was associated with the original EMPD anatomic site, including female genitourinary tract cancer associated with vulvar EMPD and gastrointestinal tract cancers in men and women associated with perianal EMPD [14]. In that study, the authors only evaluated malignancies occurring at least one year after diagnosis of EMPD so that synchronous malignancy would not be considered secondary; as such, they found that most of the increased risk was associated with anorectal malignancies. In patients diagnosed with colorectal and vulvar EMPD, increased risk reflected secondary colorectal, anal and vulvar malignancies [14]. Given this evidence, it is critical that patients with EMPD at any anatomic site receive close surveillance for the earliest possible detection of secondary malignancies. The evidence also raises questions about mammary PD and its 100% association with breast cancer: could this possibly be a secondary malignancy associated with the original anatomic site? However, Karamet al. [14] found no breast, ovarian, uterine or cervical cancers, and found no association between invasive EMPD and mammary PD, suggesting that the two entities may be fundamentally different. Pathogenesis of mammary PD and EMPD deserves further study.

### Prognostic factors for overall survival

In the present study, male gender, older age at diagnosis and races other than white and black were associated with lower overall survival, and correspondingly higher mortality. In a study of 301 patients with invasive EMPD, factors associated with survival included tumor thickness, lymphovascular invasion and number of lymph node metastases, and the presence of distant metastases [15]. Other authors also emphasize the usefulness of sentinel lymph node biopsy as a prognostic factor [16]. Although distant metastasis agrees with our results, we found no correlation between tumor size and mortality, and we did not include lymphovascular invasion. However, it must be noted that in patients without metastases, tumor thickness, that is, depth of invasion, correlates with worse survival [15].

In the SEER study, races were listed as white, black and a grouping of other races shown as Asians, Pacific Islanders, American Indian, Alaska Natives (A/PI/AI/AN), of which a considerable percentage was considered to have been Asian [8]. Asians are reported in other studies to have a four-fold higher incidence of EMPD than white patients [8, 17]. EMPD is reported to occur mainly in white women and in Asian men between ages 60 and 70 years [18]. In studies of Chinese patients, males demonstrated distinct clinical and pathological features, including genetic abnormalities and more cases of invasive EMPD, factors that would likely influence incidence, management, and prognosis [11, 19]. In the present study, OS was lowest among patients of races other than white or black and, although we cannot assume this correlates with incidence of Asian patients, we cannot rule out that cultural and/or genetic factors may play a role in frequency and severity of EMPD.

Surgery with wide local excision is reported by other authors to be the mainstay of successful treatment of EMPD at most anatomic sites [17, 20, 21], and our data show that surgery is associated with lower mortality. However, the lower mortality was only observed in patients who did not receive radiation. Possibly suggesting that patients treated with radiation tended to have more advanced disease and surgery was not able to locally control the disease. It is possible that some patients with advanced disease were only treated with radiation. Certain patients who are not candidates for surgery may continue to be treated with chemotherapy and radiation therapy, although these treatments, especially radiation, are associated with poorer outcomes [14, 17, 21]. Our results showed clearly that patients receiving radiation had poorer overall survival. This result was not entirely supported by Tolia et al. [22], who suggest that adjuvant or salvage radiotherapy may still be useful in EMPD when local recurrence, lymph node metastasis, close or positive surgical margins, large tumor size, or adenocarcinoma are present. Ito et al. [23] reported that 6% of patients develop local recurrence even after curative surgical excision, and distant metastases may develop up to two years after surgery. Also, some surgeries, such as that for perianal EMPD, present a greater surgical challenge due to characteristic depth of invasion and greater difficulty of achieving complete resection than with other surgeries that can achieve negative surgical margins [21]. This possibility may account for the higher mortality shown for EMPD of “other” anatomic sites in the present study. Authors of a study of EMPD of the groin, penis and scrotum point out the importance of family history of cancer and the absence of guidelines for treating locally advanced unresectable EMPD [24]. The absence of a staging system for invasive EMPD is also a noted drawback when evaluating this disease, as well as an obvious clinical disadvantage [15].

### Strengths and limitations

The main strength of this study was the use of a large, nationally representative sample from the United States, which minimized discrepancies and biases and allowed results to be generalized to the US adult population. The sample size was particularly helpful because, to date, most studies have been done on small case series, single centers, and using limited datasets. However, this study also has certain limitations, including that the SEER data did not show comorbidities, lifestyle and risk factors, environmental exposure, or family history, and the absence of such data may limit interpretation of factors associated with survival. The SEER data also did not include information on the use of adjuvant chemotherapy, which also could have influenced survival rates.

## Conclusions

Among EMPD patients, mortality is higher in patients with vaginal EMPD than in those with vulvar or labial EMPD and higher in those who are older, those with concurrent malignancy or distant metastasis, and higher in males than in females. Surgery is a protective factor for OS and radiation is a risk factor. Greater understanding of EMPD clinical characteristics, and the consideration of EMPD in the differential diagnosis of chronic genital and perianal dermatoses, may support early EMPD diagnosis and definitive surgical treatment.
